# Transient Downregulation of Nanog and Oct4 Induced by DETA/NO Exposure in Mouse Embryonic Stem Cells Leads to Mesodermal/Endodermal Lineage Differentiation

**DOI:** 10.1155/2014/379678

**Published:** 2014-12-03

**Authors:** Sergio Mora-Castilla, Juan R. Tejedo, Rafael Tapia-Limonchi, Irene Díaz, Ana B. Hitos, Gladys M. Cahuana, Abdelkrim Hmadcha, Franz Martín, Bernat Soria, Francisco J. Bedoya

**Affiliations:** ^1^Andalusian Center for Molecular Biology and Regenerative Medicine (CABIMER), University Pablo de Olavide, Biomedical Research Network (CIBER) of Diabetes and Related Metabolic Diseases, Red-Tercel, Avenida Américo Vespucio S/N, 41092 Seville, Spain; ^2^Sanford Consortium for Regenerative Medicine, University of California San Diego, 2880 Torrey Pines Scenic Drive, La Jolla, CA 92037, USA; ^3^Andalusian Center for Molecular Biology and Regenerative Medicine (CABIMER), Progress and Health Foundation, Biomedical Research Network (CIBER) of Diabetes and Related Metabolic Diseases, Red TerCel, Américo Vespucio S/N, 41092 Seville, Spain

## Abstract

The function of pluripotency genes in differentiation is a matter of investigation. We report here that Nanog and Oct4 are reexpressed in two mouse embryonic stem cell (mESC) lines following exposure to the differentiating agent DETA/NO. Both cell lines express a battery of both endoderm and mesoderm markers following induction of differentiation with DETA/NO-based protocols. Confocal analysis of cells undergoing directed differentiation shows that the majority of cells expressing Nanog express also endoderm genes such as Gata4 and FoxA2 (75.4% and 96.2%, resp.). Simultaneously, mRNA of mesodermal markers Flk1 and Mef2c are also regulated by the treatment. Acetylated histone H3 occupancy at the promoter of Nanog is involved in the process of reexpression. Furthermore, Nanog binding to the promoter of Brachyury leads to repression of this gene, thus disrupting mesendoderm transition.

## 1. Introduction

Embryonic stem cells (ESCs) self-renew continuously and differentiate into any cell type. Mouse ESCs self-renewal is at least in part controlled by extracellular signals such as leukemia inhibitory factor (LIF), which maintain the undifferentiated state through activation of Stat3 pathway [1]. Studies over the past few years have revealed the role played by transcription factor networks and epigenetic processes in the maintenance of ESC pluripotency [2]. Current evidence suggests that ESC pluripotency is orchestrated by the expression of Nanog, Oct4, Sox2, and Ronin genes [2–7]. Nanog plays a prominent role in the maintenance of pluripotent epiblast and in the prevention of differentiation towards primitive endoderm of cells of inner mass during embryonic development [4]. On the other hand, heterogeneous expression of Nanog has been documented in ESCs and fluctuations in Nanog expression in cells are being suggested to provide an opportunity for differentiation [8–10]. Furthermore, Nanog overexpression blocks ectoderm and definitive endoderm (DE) differentiation in both human ESCs and mouse epiblast cells [11]. Additional roles of these genes might be considered since they are also expressed in DE cells [12–15].

Changes in chromatin structure also play a role in the biology of stem cells [16–18]. Through epigenetic processes, the pluripotent epigenome keeps the chromatin structure open to allow for rapid genetic regulation [19]. Nitric oxide (NO) is a signalling molecule that plays a role in developmental processes, but the mechanisms involved are still a matter of study [20–22]. In this regard, differentiation of ESC into cells derived from the three embryonic germ layers following* in vitro* exposure to NO has been reported [23–26]. In addition, global changes in histone acetylation during induction of endodermic [23] and mesodermic [27] differentiation by NO have been described. These findings highlight the relevance of combined actions of signalling pathways and epigenetic rearrangements during ESC differentiation. In this study, we report that downregulation of stemness transcription factors during NO-induced differentiation is transient and that occupancy of promoter regions of differentiation genes such as Brachyury by these factors plays a role in the control of lineage specification.

## 2. Materials and Methods

### 2.1. Cell Culture, DETA/NO Treatment, Definitive Endoderm Differentiation, Transfection, and Sorting

D3 mESCs (ES-D3 ATCC CRL-1934) were cultured at 37°C with 5% CO_2_. Cells were maintained in Dulbecco's modified Eagle's medium (DMEM) (Gibco, Paisley, UK), supplemented with 15% heat-inactivated fetal bovine serum (FBS) (Hyclone, Logan Utah, USA), 0.5 mM *β*-mercaptoethanol (Gibco), 2 mM L-glutamine (Gibco), 0.1 mM MEM Non-Essential amino acids (Gibco), 5000 U/mL penicillin/streptomycin (Gibco), and 1000 U/mL LIF (ESGRO, Chemicon, Billerica, MA, USA). When appropriate, cells were cultured for 3 days either in the presence or absence of LIF. Treatment with 1 mM diethylenetriamine/nitric oxide adduct (DETA/NO) (Sigma-Aldrich, St. Louis MO, USA) was carried out for 19 h in cultured medium containing 15% FBS and LIF as required. For immunochip analysis, cells were exposed to 0.5 mM DETA/NO instead. This concentration provoked lower levels of cell death than 1 mM, thus allowing obtaining sufficient amounts of nuclear protein to perform the chromatin immunoprecipitation procedure. Previous assays performed in our laboratory showed no substantial differences in the differentiating effect of the two concentrations of DETA/NO used. Cells were subsequently cultured in complete medium for an additional period of 2 days either in the presence or absence of LIF. To induce differentiation towards definitive endoderm (DE), cells were preconditioned for 3 days in the absence of LIF and subsequently exposed to 1 mM DETA/NO for 19 hours. Cells were then maintained in culture medium supplemented with 15% FBS in the absence of LIF for subsequent days. Culture medium was replaced daily in all experimental conditions.

A D3 line expressing GFP driven by Oct4 promoter (D3-Oct4 line) was generated by using 4 *μ*g of plasmid pOct-eGFP, according to a previous publication [28]. Cells were electroporated (800 V, 50 *μ*F, 30 ms) and selected by puromycin (Sigma-Aldrich) resistance and GFP expression. GFP-positive and negative cells were sorted by FACS in an Advantage SE System (BD FACS Aria Cell Sorter, BD Biosciences, Erembodegem, Belgium). D3-Oct4 cells were cultured as above. GFP cell fluorescence was visualized with an Olympus IX70 inverted microscope.

### 2.2. RNA Isolation, Reverse Transcription, and Real-Time PCR Analysis

Total RNA was extracted using TRIzol (Invitrogen, Paisley, UK) and chloroform/isopropanol purification procedure. cDNA synthesis was performed with 1 *μ*g of total RNA using M-MVL reverse transcriptase (Promega, Madrid, Spain) and random primers according to manufacturer's instructions. mRNA levels were measured by real-time PCR analysis based on SYBR Green (Applied Biosystems, Paisley, UK) and detection with ABI Prism 7500 (Applied Biosystems). Results were normalized with *β*-actin. Real-time PCR forward (F) and reverse (R) primers used are as follows:

 Nanog F: AGCAGATGCAAGAACTCTCCTCCA,
 Nanog R: CCGCTTGCACTTCATCCTTTGGTT,
 Oct4 F: AGCTGCTGAAGCAGAAGAGGATCA,
 Oct4 R: AACACCTTTCCAAAGAGAACGCCC,
 Pdx1 F: AGCTCCCTTTCCCGTGGATGAAAT,
 Pdx1 R: TAGGCAGTACGGGTCCTCTTGTTT,
 FoxA2 F:TCAACGACTGCTTTCTCAAGGTGC,
 FoxA2 R: TTCTCGAACATGTTGCCCGAGTCT,
 Gata 4 F: AGGGTGAGCCTGTATGTAATGCCT,
 Gata 4 R: AGGACCTGCTGGCGTCTTAGATTT,
 Cxcr4 F: TGATGCCATGGCTGACTGGTACTT,
 Cxcr4 R: AACGCTGCTGTAGAGGTTGACAGT,
 Hnf1*β* F: ACAGGGCAGAATGTTTGCAACGAG,
 Hnf1*β* R: TATAGGCATCCATGGCCAGCTTCT,
 Sox17 F: TTATGGTGTGGGCCAAAGACGAAC,
 Sox17 R: TCAACGCCTTCCAAGACTTGCCTA,
 
*β*-actin F: TCCTGTGGCATCCACGAAACTACA,
 
*β*-actin R: ACCAGACAGCACTGTGTTGGCATA.

### 2.3. Protein Extraction and Western Blot

Proteins were extracted with RIPA buffer (Sigma-Aldrich) supplemented with a cocktail of protease and phosphatase inhibitors (Sigma-Aldrich). Briefly, cells were trypsinized from culture dishes, centrifuged, and washed once with cold PBS. Cell pellets were then resuspended and incubated in RIPA buffer for 20 min on ice and sonicated with 3 pulses 10 sec each at 10% amplitude (Branson Digital Sonifier, Danbury, CT, USA). After centrifugation, supernatants were denatured in Laemmli loading buffer for 10 minutes at 98°C. Total protein (20 *μ*g) was separated by SDS-PAGE and transferred to PVDF membranes which were subsequently probed with anti-Nanog (Bethyl, Montgomery TX, USA), anti-Oct4 (BD Transduction, San José, CA, USA), anti-Pdx1 (Abcam, Cambridge, UK), anti-Sox17 (Millipore, Billerica, MA, USA), or anti-Gata4 (Santa Cruz Biotechnology, Dallas, TX, USA), and anti-*β*-actin (Sigma-Aldrich) as loading control.

### 2.4. Immunofluorescence

For confocal studies, cells were seeded on coverslips as follows. Glass coverslips 24-mm in diameter were washed in hydrochloric acid and sonicated in a water bath for 30 min. Coverslips were subsequently rinsed with 50% ethanol and sonicated for 30 min. Following washes with 70% and with 95% ethanol and sonication, coverslips were air-dried and placed in 6-well tissue culture plates (Nunc, Rochester, NY, USA). Coating with Matrigel (BD Biosciences, California, USA) for 2 hours at 37°C ensues. Next, coverslips were washed with Knockout Dulbecco's modified Eagle's medium (Gibco) and seeded with D3 cells at a density of 75 × 10^3^ cells/well. Cells were cultured and induced to differentiate as above. Then, cells were fixed with 4% paraformaldehyde for 10 min, followed by permeabilization with methanol (−20°C) for 5 min. Samples were then exposed to blocking solution for 30 min (3% BSA in PBS + 0.1% Tween 20) and incubated overnight at 4°C with rabbit anti-Nanog (Bethyl Laboratories, Montgomery, USA), mouse anti-Gata4 (Santa Cruz Biotechnology), and goat anti-FoxA2 (Santa Cruz Biotechnology). For detection of primary antibodies, anti-mouse Alexa Fluor 488 (Invitrogen, Canada), anti-rabbit Alexa Fluor 568 (Invitrogen), and anti-goat Alexa Fluor 488 (Invitrogen) were used. Cells were counterstained with 300 nM DAPI (Tocris, Bristol, UK). Coverslips were mounted on microscope slides with VECTASHIELD mounting medium (Vector Laboratories, Burlingame, California) and visualized with a confocal microscope (Leica TCS SP5) using a HCX PL APO Lambda Blue 100 × 1.4 oil immersion objective. For quantification, 101 cells with one (Nanog or Gata4) or two (Nanog and Gata4) stains from 9 different fields and 116 cells with one (Nanog or FoxA2) or two (Nanog and FoxA2) stains from 7 different fields from one experiment were recorded.

### 2.5. Chromatin Immunoprecipitation Assay

Batches of 3 × 10^6^ cells were cross-linked with 1% (w/v) formaldehyde for 10 min at 37°C. Cells were then resuspended in lysis buffer containing 10 mM Tris HCl (pH 8.0), 10 mM NaCl, 3 mM MgCl_2_, 0.5 mM DTT, and protease inhibitors for 10 min on ice. Following centrifugation at 4°C for 5 min, supernatants were discarded and pellets were washed by gentle inversion with 10 mM Tris HCl buffer, pH 8.0 containing 15 mM NaCl, and 60 mM KCl and centrifuged for 5 min at 4°C. Pellets were then resuspended in washing buffer supplemented with 3 mM CaCl_2_, protease inhibitors, 0.5 mM DTT, and 5–10 *μ*L of 1 : 200 micrococcal nuclease (New England BioLabs) and incubated for 20 min at 37°C with orbital shaking. Nuclease activity was halted by addition of 20 *μ*L 0.5 mM EDTA and preparations were then centrifuged for 5 min at 3000 rpm. Supernatants were discarded and pellets were resuspended in buffer containing 150 mM NaCl, 50 mM Tris HCl (pH 7.5), 5 mM EDTA, 0.5% NP-40, 1% Triton, and 0.01% SDS and sonicated with 3 pulses, 10 s each at 10% amplitude. Sonicates were then centrifuged for 10 min at 10000 rpm at 4°C, and supernatants containing chromatin extracts with an average size of 500 bp were immunoprecipitated with 2–4 *μ*g of each specific antibody per sample. Anti-acetyl histone H3 (Abcam), anti-Nanog (Bethyl) were used. Anti-Rabbit IgG (Abcam) was used as mock ChIP control. Then, 30 *μ*L of blocked Dynabeads (Invitrogen, Dynal AS, Oslo, Norway) were used to prepare antibody-bead complexes and incubated for 1 h at 4°C under rotation in dilution buffer (0.01% SDS, 1.1% Triton X-100, 1.2 mM EDTA, 16.7 mM Tris HCl (pH 8.1), 167 mM NaCl). Then, chromatin was added and incubated for 1 hour under the same conditions. Complexes were washed once with low-salt buffer (0.1% SDS, 1% Triton X-100, 2 mM EDTA, 20 mM Tris HCl pH 8.1, 150 mM NaCl), once with high-salt buffer (0.1% SDS, 1% Triton X-100, 2 mM EDTA, 20 mM Tris HCl pH 8.1, 500 mM NaCl), once with LiCl buffer (0.25 M LiCl, 1% NP40, 1% Deoxycholate, 1 mM EDTA, 10 mM Tris HCl, pH 8.0), twice with TE buffer (10 mM Tris HCl, pH 8.0, 1 mM EDTA) and finally eluted with 500 *μ*L solution containing 1% SDS and 0.1 M NaHCO_3_. DNA purification was performed with phenol : chloroform procedure. ChIP analysis was carried out by real time-PCR using SYBR Green. Promoter occupancy was determined by percent input method.

Primers used were as follows:

 Nanog promoter F: CCCTAAGCTTTCCCTCCCTCC,
 Nanog promoter R: CCAAATCAGCCTATCTGAAGG,
 Brachyury promoter NngBSu F: CCAGAGGTTGGCTCCTGGAAA,
 Brachyury promoter NngBSu R: GGTGTGGGCAGCAGTTGGTTTATT,
 BrachP F: TTGCGGGAGTTCAAGTGGAGC,
 BrachP R: CTCTCCACCTTCCAGGAGTCTTGA.

### 2.6. Statistical Analysis

Data shown are expressed as mean ± SEM of three independent biological replicates unless otherwise indicated. Student's *t*-test was used. A value of *P* < .05 was accepted as statistically significant.

## 3. Results

### 3.1. Nanog and Oct4 Are Reexpressed following NO-Induced Differentiation

**Figure 1:**
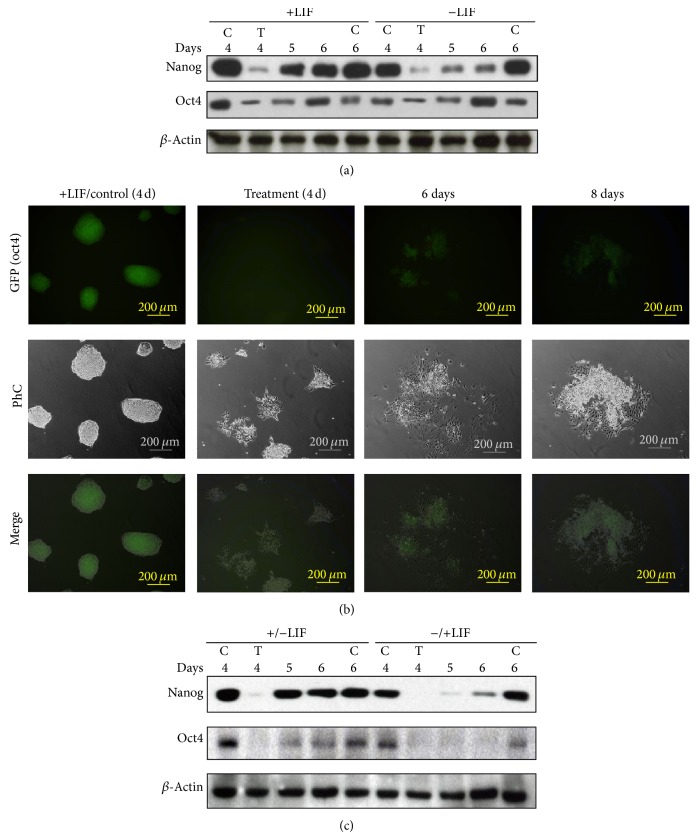
Reexpression of pluripotency genes following exposure to DETA/NO. (a) Western blot analysis of Nanog and Oct4 proteins. D3 mESCs were grown in the presence (+LIF) or in the absence (−LIF) of LIF for 3 days. Cells were then cultured as indicated in Section 2 for additional periods of 1 and 2 days (day 4 and day 5). C: control cells. T: cells exposed to 1 mM DETA/NO for 19 h on day 4. Lanes 5 and 6 refer to cells exposed to DETA/NO and subsequently cultured for 1 and 2 days as in Section 2. (b) GFP expression in D3-Oct4 mESCs. Control cells were cultured in the presence of LIF for 6 days (+LIF/Control). Treated cells were cultured in the presence of 1 mM DETA/NO for 19 hours on day 4 and subsequently cultured as indicated in Section 2 for an additional period of 2 days in absence of LIF. PhC: phase contrast. Results are representative of 3 independent experiments. (c) Western blot analysis reveals Nanog and Oct4 reexpression after DETA/NO treatment. +/− LIF indicates that cells were cultured for 4 days in the presence of LIF, then LIF was removed and cells were subsequently exposed to 1 mM DETA/NO for 19 hours. Days 5 and 6 indicate that cells were subsequently cultured for 1 and 2 days in the absence of LIF. −/+LIF indicates cells that were cultured for 4 days in the absence of LIF; exposure to 1 mM DETA/NO for 19 hours was then performed in the presence of LIF. Days 5 and 6 indicate that cells were subsequently cultured for additional periods of 1 and 2 days, respectively, in the presence of LIF. Results are representative of 3 independent experiments.

D3 mESCs were cultured for 3 days with or without LIF and then exposed to 1 mM DETA/NO for 19 hours (day 4). A substantial reduction in Nanog and Oct4 protein level was observed in cells following treatment with DETA/NO (Figure 1(a)). To explore the stability of Nanog and Oct4 downregulation, recovery experiments were carried out. After DETA/NO treatment, cells were grown in complete medium until day 6 in the presence or absence of LIF. The results show that Nanog and Oct4 proteins were reexpressed following DETA/NO exposure. In the case of Nanog, the phenomenon was more apparent in the presence of LIF. A similar response was observed in D3 mESCs expressing the green fluorescence protein (GFP) under the control of the Oct4 promoter. Cell colonies with homogeneous fluorescence were formed when D3 cells were cultured in the presence of LIF (+LIF/control) (Figure 1(b), GFP Oct4). Exposure to DETA/NO led to loss of GFP fluorescence in cells cultured without LIF (Treatment). Similar results were observed in cells cultured in the presence of LIF (not shown). Loss of homogeneous colony morphology is also apparent upon DETA/NO treatment (Figure 1(b), Treatment Ph C). Heterogeneity in the recovery of GFP signal is apparent following 2 days of culture in complete medium even in the absence of LIF (day 6) and up to 4 days (day 8) (see Supplementary Figure 1 in Supplementary Material available online at http://dx.doi.org/10.1155/2014/379678) since clusters of GFP positive cells coexist with GFP negative cells (see PhC image). In addition, colony morphology is not recovered.

### 3.2. Nanog and Oct4 Reexpression Is Independent of LIF

In order to explore the role of LIF on Nanog and Oct4 reexpression after DETA/NO challenge, experiments were carried out in which LIF was added or removed to/from cells preconditioned in the absence or presence of LIF, respectively, for 3 days. Results show that addition of LIF to media after NO treatment did not restore completely Nanog and Oct4 protein levels (Figure 1(c), −/+LIF conditions). Nevertheless, kinetics of Nanog and Oct4 recovery appears to be faster in cells preconditioned in the presence of LIF before DETA/NO treatment (Figure 1(c), +/− LIF conditions). Hence, LIF seems to be dispensable for Nanog and Oct4 reexpression, although previous exposure to the factor accelerates the process.

### 3.3. Histone H3 Acetylation Participates in Nanog Reexpression after NO-Induced Differentiation

**Figure 2:**
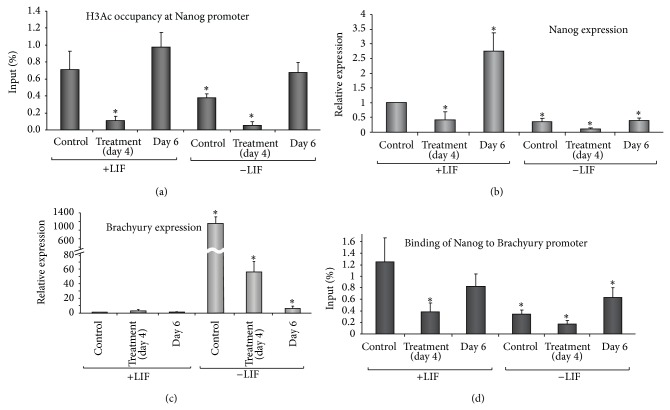
Nanog reexpression during differentiation acts as a regulator of cell fate decisions (a) ChIP analysis shows relative levels of acetylated histone H3 occupancy at Nanog promoter following NO treatment. (b) Real-time PCR analysis of Nanog and (c) Brachyury expression levels before and after NO treatment. (d) ChIP analysis shows Nanog binding to Brachyury promoter following NO treatment. Histogram plots represent cells from control, NO treated (day 4), and 2 days after NO treatment (day 6) in the presence (left bars) or absence (right bars) of LIF, respectively. Data are mean ± SEM from 5 independent biological replicates.  ^*^Significant difference from undifferentiated control condition (+LIF).

Previous reports have shown that NO induces chromatin reorganizations in mESCs [25, 27]. Thus, we studied changes in the levels of acetylated histone occupancy at the Nanog promoter (Figure 2(a)). Chromatin immunoprecipitation studies show occupancy of acetylated histone H3 on Nanog promoter in control cells cultured in the presence of LIF. A significant reduction was observed in cells cultured in the absence of LIF for the same period of time (Figure 2(a)). The DETA/NO treatment led to a strong and significant reduction in occupancy, either in the absence or presence of LIF. Occupancy is fully recovered 2 days after exposure to DETA/NO. This phenomenon is more apparent in the presence of LIF. Occupancy at Nanog promoter by acetylated histone H3 parallels the Nanog reexpression after DETA/NO treatment both at mRNA (Figure 2(b)) and protein (Figure 1(a)) in cells cultured with LIF. The fact that no substantial recovery of Nanog mRNA levels is observed in cells grown in the absence of LIF indicates that LIF signaling is required for the expression of this gene.

In order to study the role of Nanog in differentiating cells, we analyzed the expression of Brachyury, a reputable mesendoderm marker under the control of Nanog. A strong and significant expression of Brachyury was observed in cells grown in the absence of LIF when compared with cells cultured in the presence of LIF. The exposure to DETA/NO leads to significant decrease in Brachyury expression in LIF-deprived cells. Brachyury repression is more pronounced 2 days after DETA/NO treatment (Figure 2(c)). Consistently with its role as repressor, Nanog occupancy at Brachyury promoter was significantly diminished in cells cultured under −LIF conditions and the exposure to DETA/NO decreases it even more (Figure 2(d)). Occupancy was restored to control levels 2 days after DETA/NO treatment in cells grown in the presence of LIF and this coincides with Brachyury repression. DETA/NO treatment led a significant decrease of Nanog binding to Brachyury promoter in cells grown in the presence of LIF. A nonsignificant rise in Brachyury mRNA in this condition suggests that additional factors are involved in the repression of this gene. Enhanced binding of Nanog at Brachyury promoter accompanies a strong repression of the gene after DETA/NO treatment (Figures 2(c) and 2(d)). It is further observed that changes in the acetylation pattern at Brachyury promoter participates in the regulation of its mRNA expression. There is a strong occupancy signal at the promoter in −LIF condition according to the mRNA expression. NO treatment induces a decrease on the promoter occupancy consistent with the significant decrease of gene expression (Supplementary Figure 2). According to the activation of H3 acetylated, occupancy at the Brachyury promoter is also observed in +LIFNO condition. This, in concert with low Nanog occupancy (Figure 2(d)) could account for the regulation of Brachyury expression.

### 3.4. Differentiated Cell Populations Express Definitive Endoderm Markers in addition to Nanog Reexpression

**Figure 3:**
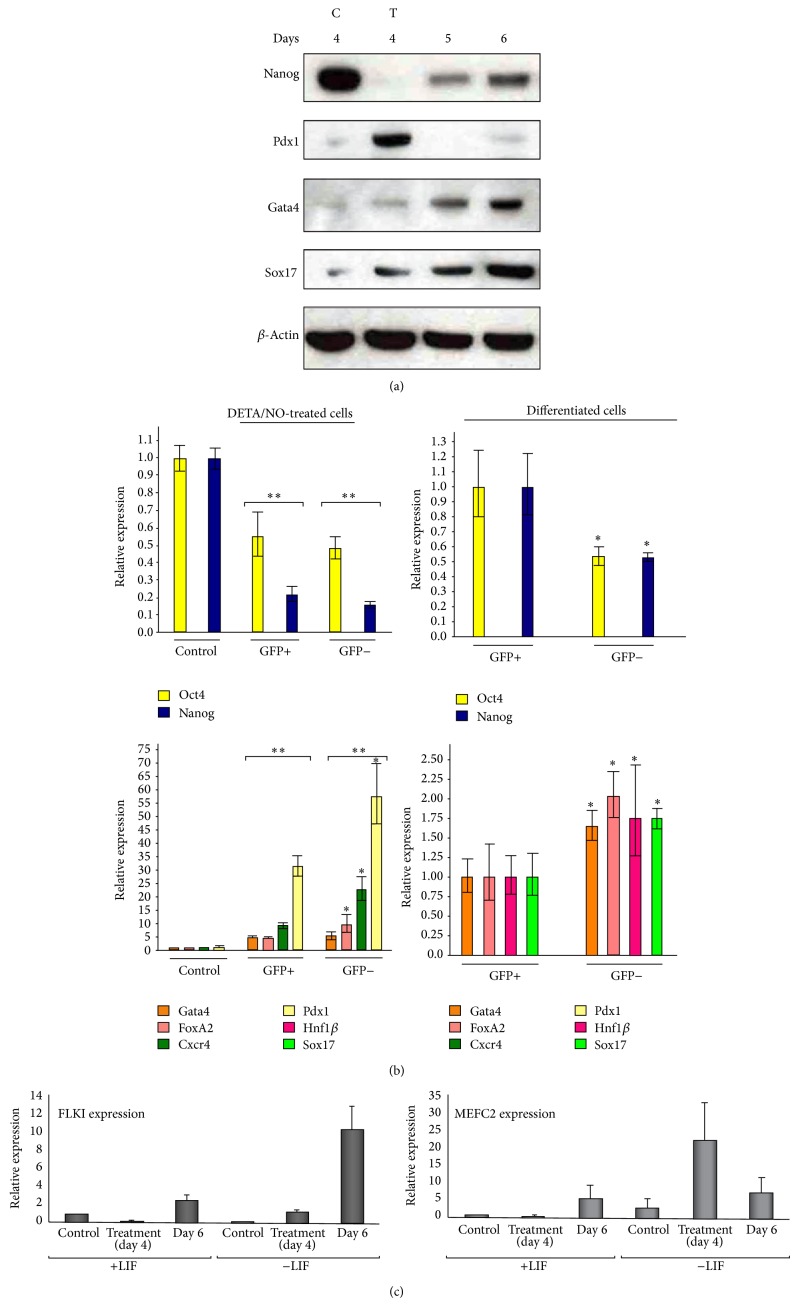
Nanog is expressed in differentiated cells. (a) Western blot analysis shows endoderm markers and Nanog reexpression after NO treatment. Blots are representative of three independent experiments. C: control cells cultured with LIF for 4 days; T: cells cultured with LIF for 3 days and then exposed to 1 mM DETA/NO for 19 h. Days 5 and 6 refer to DETA/NO treated cells cultured in the absence of LIF for additional periods of 1-2 days. (b) Endoderm markers are expressed in both Oct4 positive and negative cell populations after* in vitro* differentiation. Real-time PCR analysis for expression of pluripotent (Oct4 and Nanog) and endoderm (Gata4, FoxA2, Cxcr4, Pdx1, Sox17) markers in control (undifferentiated cells grown for 4 days in the presence of LIF), treated and DE differentiated GFP positive and GFP negative cells. D3-Oct4 cells were grown, differentiated, and sorted for GFP expression according to the protocol described in Section 2. Control cells indicate cells grown for 4 days in the presence of LIF; NO-treated cells indicate cells maintained for 3 days in the absence of LIF and treated for 19 h with 1 mM DETA/NO. DE-differentiated cells indicate cells 2 days after treatment in the absence of LIF.  ^*^Significant difference from GFP^+^;  ^**^significantly different from control condition. (c) Quantitative real-time PCR for mesodermal lineage markers expressed before and after DETA/NO treatment.

In order to assess the impact of Nanog and Oct4 reexpression on the differentiation process, the expression of definitive endoderm (DE) markers was analyzed in a defined protocol for generation of DE cells.  Figure 3(a) shows a remarkable downregulation of Nanog and an increase in Pdx1 expression upon DETA/NO treatment in mESC. In addition, differentiated cell populations reexpress Nanog and increase expression of DE factors such as Gata4 and Sox17 (Figure 3(a)) 1 and 2 days after NO treatment. Pdx1 expression levels, however, decrease after NO treatment (Figure 3(a)). To assess the relevance of Oct4 reexpression on upregulation of DE markers, a mESC line expressing GFP under the control of Oct4 promoter was used. Following differentiation, sorted GFP positive and GFP negative cells were analyzed for expression of Nanog, Oct4, and different endoderm markers (Figure 3(b)). NO treatment induces downregulation of Oct4 and Nanog and upregulation of endoderm markers Gata4, FoxA2, Cxcr4, and Pdx1 in both GFP negative cells and in residual GFP positive cells (Figure 3(b), upper and lower left graphs). Only two days after exposure to differentiating stimulus, both GFP positive and negative cells upregulate Gata4, FoxA2, Hnf1*β*, and Sox17, with significantly higher levels in GFP negative cell population (Figure 3(b), upper and lower right graphs). In addition, in order to help understand the differentiation phase, mesodermal markers Flk1 and Mef2c were analyzed by quantitative real-time PCR. In the presence of LIF the expression of both markers decreases following exposure to DETA/NO, and upregulates after 2 days. On the other hand, in absence of LIF the treatment induces Flk1 expression and maintains the increase for 2 days more; nevertheless, Mef2c also increases its expression after NO but the increase is not sustained at day 6 (Figure 3(c)).

**Figure 4:**
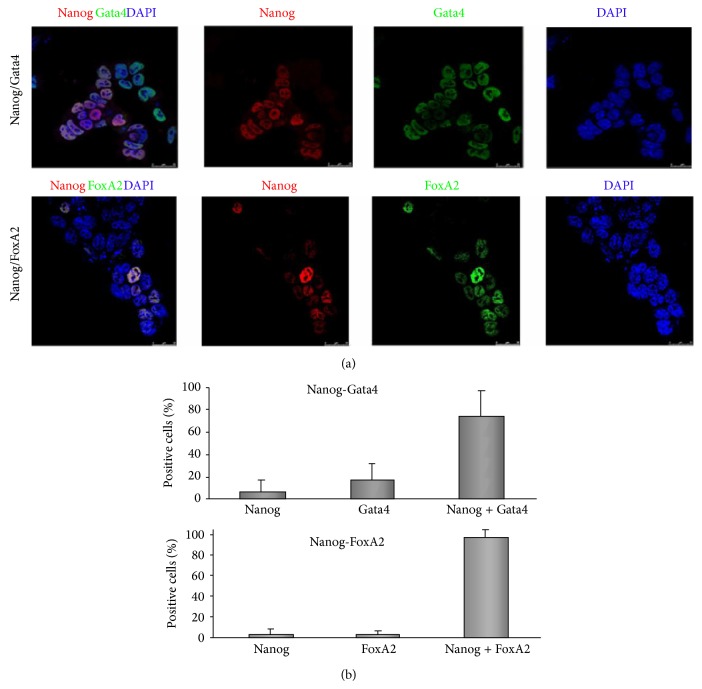
Nanog colocalizes with Gata4 and FoxA2 in definitive endoderm cells. Cells were differentiated according to protocol described in Section 2 and analysed by confocal immunofluorescence (a) for Nanog, Gata4, and FoxA2 at day 8 of differentiation. (b) Analysis showing the percentage of cells coexpressing Nanog-Gata4 and Nanog-FoxA2 in D3 differentiated cells. Data are mean ± ESM from 7–9 fields.

To test the possibility that the presence of stemness genes in differentiated cell populations might indeed be ascribed to cells escaping from differentiation processes, confocal microscopy immunofluorescence analyses were performed in cells induced to differentiate into DE. Nanog expression increases from 0.65% (data not shown) in NO-treated cells to 54% in differentiated cells population (data calculated from Figure 4(a) ratio Nanog + versus Dapi). Results show that Nanog is heterogeneously expressed in DE-differentiated cells (Figure 4(a)) and that the majority of Nanog positive cells also coexpressed Gata4 and FoxA2 (75.4% and 96.2%, resp.), with the cells expressing only Nanog no more than 7% (Figures 4(a) and 4(b)).

## 4. Discussion

The role of genes such as Nanog and Oct4 in orchestrating the pluripotent state of ESC is well established. Their potential role in differentiated states remains, however, to be fully validated [8–10]. Previous studies have shown that Nanog downregulates transiently in mESC cultured in homogeneous conditions in the presence of LIF [8–10]. Fluctuations in expression levels of Nanog were also described in cells of the embryo inner mass and in germ cells of developing embryos [9]. Although the pluripotent state of mESC is maintained* in vitro* by LIF in a Nanog dependent manner, little information is available on the mechanism involved in the control of its transient expression [1]. We show here that Nanog reexpression after NO-induced downregulation is LIF-independent. Interestingly, previous culture of cells in the presence of LIF for 4 days allows substantial recovery of Nanog and Oct 4 (+/−LIF condition) when compared with cells cultured in the absence of LIF (−/+LIF condition) (Figure 1(c)); thus reexpression is more efficient in cells previously preconditioned in the presence of LIF. In summary, the use of LIF in the reexpression experiments shows that although Nanog and Oct 4 reexpression after NO challenge does not require LIF, previous exposure to this factor is instrumental for this response, thus suggesting that LIF-dependent factors stabilize the pluripotency network.

Otherwise, after NO treatment Nanog is strongly downregulated at the protein and transcript level in the presence and absence of LIF. However, in the presence of LIF, 2 days after NO treatment, levels of Nanog transcript are much higher than expected at the protein level. This phenomenon makes us think that Nanog epigenetic context is different depending on LIF presence or absence during pharmacological treatments with DETA-NO. Moreover, strong occupancy of acetylated histone H3 of the Nanog promoter was observed 2 days after NO treatment both in the presence and absence of LIF, thus setting the stage for reactivation of this pluripotency gene. Global epigenetic changes also occur during NO-induced differentiation and some of these epigenetic modifications are maintained after withdrawal of the differentiating factor [23]. On the other hand, transient suppression of Nanog following treatment of mESC with the histone deacetylase inhibitor TSA (Trichostatin A) has been reported to be associated with deacetylation of Nanog promoter region and with start of differentiation [29]. We report here enhanced occupancy of acetylated histone H3 at Nanog promoter two days after withdrawal of the differentiating stimulus. This phenomenon is more apparent in the presence of LIF, coincident with strong Nanog reactivation. All these data suggest that specific epigenetic modifications of chromatin after NO-treatments finely regulate* in vitro* reactivation of Nanog.

The scenario of gene regulation that is proposed here for mESC differentiation by NO treatment goes through the disruption of mesendoderm transition by silencing of Brachyury and rising the expression of mesoderm and endoderm markers. It has been reported that Brachyury regulation is controlled by histone acetylation at its promoter [30], supporting our data in which NO treatment induces changes in the occupancy of H3 acetylated, increasing in the presence of LIF and decreasing in absence of LIF; this action is suggested to be complementary with the decrease of the repressive action of Nanog. This effect on the gene regulation of Brachyury suggests a selective activity of NO treatment in the differentiation transition towards mesoderm and endoderm lineages. The specification of lineage commitment after Nanog and Oct4 downregulation is a matter of debate. Previous reports have shown differentiation towards primitive endoderm [8, 31] and mesoderm [27]. In addition, Nanog null cells do not differentiate solely into primitive endoderm, showing multilineage differentiation capacity [9]. To assess the differentiating capacity and commitment of differentiated cells in our experimental design, beside the expression of endodermal genes, we analyzed the expression of Brachyury and mesodermal genes Flk1 and Mef2c. NO treatment led to substantial and sustained decrease of Brachyury and enhanced expression of endoderm markers Pdx1, Gata4, and Sox17, reflecting thus an endoderm selective process, although the upregulation of Flk1 and Mef2c is evident suggesting also the generation of mesoderm lineage. In fact, global epigenetic context is different before and after NO treatment, creating a differentiating scenario after NO stimulus with a transient downregulation of Nanog and a disruption of the mesendodermal transition. This notion has received support from studies with human ESCs and with different protocols. In fact, previous studies from Lie et al. [32] observed that Nanog suppression in human ESCs causes upregulation of DE genes and efficient differentiation to pancreatic endoderm, confirming our observations. Nevertheless, Nanog [15] and Oct4 [12, 13] are expressed in DE progenitors after differentiation with activin A, reinforcing our observations using NO as a differentiating agent. Furthermore, it has been reported that pharmacological blockade of Activin/Nodal signalling pathway during DE differentiation prevents proper commitment of human ESCs to the endodermal lineage [14]. Jaremko and Marikawa showed a reduction in the Oct4 and Nanog expression levels after 2 days of Nodal/Activin signaling inhibition. Nevertheless, Nanog transcripts were reactivated after activin A addition. In fact, the levels of Oct4 and Nanog transcripts were not decreased during the first 6 days of DE differentiation in culture [14], resembling similar results that we described here. In our model of DE differentiation, we selected Oct4 reexpressing cells (GFP positive) and analyzed them for expression of endoderm markers. Both GFP positive and negative cell populations increase the expression of Gata4, FoxA2, and Sox17 during differentiation. GFP-negative cell population had higher levels of expression of DE markers, according to its more differentiated morphology. After NO treatment in LIF starving conditions, few cells (around 3% of the population) still express GFP at the protein level, but this is not related to significant changes in the transcript level of Nanog between GFP positive and negative cells. However, two days after NO treatment Nanog transcript is slightly lower in GFP negative population compared with GFP positive cells in DE-cell population. This phenomenon can be attributed to different half-life ratios of GFP-protein and Nanog-transcript. The fact that Nanog and Oct4 are reexpressed heterogeneously after NO treatment and that Nanog coexpresses with Gata4 and FoxA2 in cells gives support to the notion that the protocol used here generates terminally differentiated DE cells.

**Figure 5:**
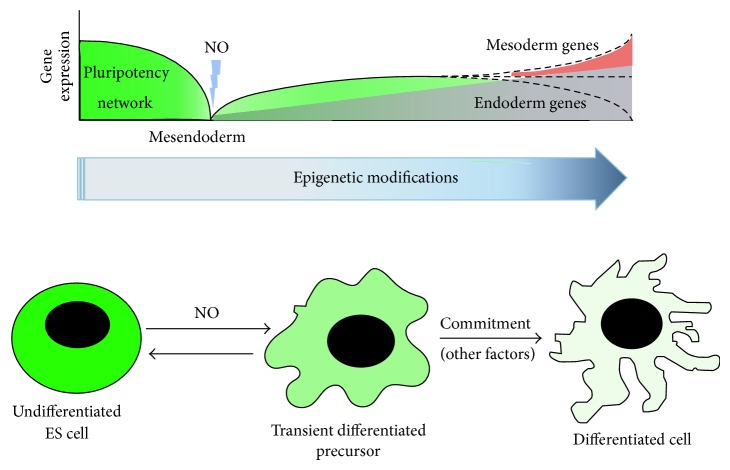
Model for stem cell differentiation. During ES cell differentiation, Nanog and Oct4 expression is downregulated and nascent epigenetic modifications occur. At this point, nitric oxide can participate in transient downregulation of pluripotency network and also in chromatin remodelling events. Nevertheless, Nanog can be reexpressed in transient differentiated precursors. By this, fluctuating levels of Nanog and Oct4 constitute an “opportunity” for commitment through specific lineage differentiation. Nitric oxide participates in this perturbation, but further intrinsic or environmental stimuli are needed for correct commitment decision.

All in all, the data reported here indicate that pluripotency factors play additional roles other than controlling the machinery of pluripotency. In fact, Oct4 has a dual role working with Sox2 to maintain pluripotency and with Sox17 to promote endodermal lineage differentiation [30]. In our case, environmental perturbations caused by high levels of NO provoke Nanog and Oct4 downregulation and epigenetic chromatin modifications, constituting an opportunity for subsequent differentiation (see Figure 5). Heterogeneous reexpression of Nanog or Oct4, however, is not a barrier for DE differentiation, but an inherent property of mESC. This mechanism might be an evolutionary selected strategy to respond and adapt to the markedly changing external environment and for the generation of new progenitors during differentiation. In fact, during the implantation of the blastocyst, inducible NO synthase (iNOS) levels increase markedly in the uterine tissue of rodents [33]. This phenomenon correlates with the transient decrease in Nanog in newly implanted blastocysts [4, 34]. Subsequently, Nanog is reexpressed in epiblast cells [35], simulating the schemes described in this work. Thus, one would expect the relevance of NO signalling as a new biological system regulating the pluripotency in embryonic cells, setting the stage for the development of new strategies for regenerative medicine.

## 5. Conclusions

Nitric oxide has been used as a differentiator agent in both mouse and human ESCs. The process involves transient downregulation of master genes of pluripotency such as Nanog and Oct4. The present paper shows that Nanog reexpression after NO-induced differentiation involves epigenetic changes on Nanog promoter. Recruitment of Nanog on Brachyury promoter blocks the reactivation of this mesendodermal marker. Definitive endoderm differentiated cells coexpress endoderm markers and Nanog and the expression of mesodermal genes is also apparent. It is thus proposed that reexpression of Nanog and Oct4 plays a role in the differentiation towards specific cell lineages.

## Supplementary Material

The Supplementary Material shows the dynamics of reexpression of Nanog and Oct4 protein after DETA/NO challenge. In addition, a ChIP assay was performed to identify the occupancy of the H3 histone acetylated at Brachyury promoter in order to determine the regulation of this gene by nitric oxide. Antibodies and methodology used for both experiments is described in Section 2.
