# Supplementary material for: Transient Downregulation of Nanog and Oct4 Induced by DETA/NO Exposure in Mouse Embryonic Stem Cells Leads to Mesodermal/Endodermal Lineage Differentiation
Source: Stem Cells Int. 2014 Dec 3;2014:379678. doi: 10.1155/2014/379678 (PMC4269087; doi:10.1155/2014/379678)

Supplementary figure 1. Re-expression of pluripotency genes following exposure to DETA/NO. Western blot analysis of Nanog and Oct4 proteins. D3 mESCs were grown in the presence (+LIF) or in the absence (-LIF) of LIF for 3 days. Cells were then cultured as indicated in *Materials and Methods* for additional periods of 2 and 4 days (day 6 and day 8). C: control cells. T: cells exposed to 1mM DETA/NO for 19 h on day 4. Lanes 5 and 6 refers to cells exposed to DETA/NO and subsequently cultured for 2 and 4 days as in *Materials and Methods*

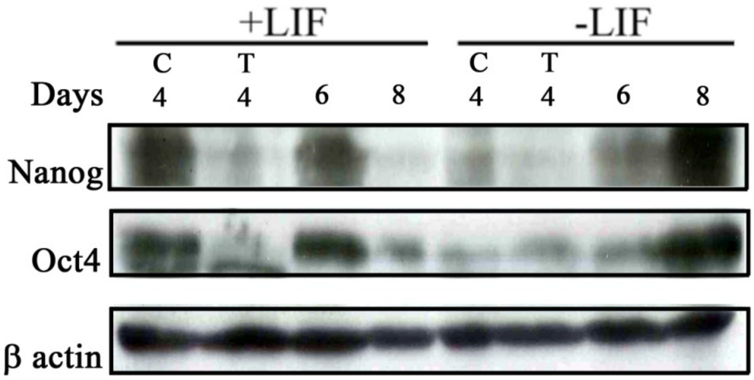

Supplementary figure 2. Acetylation of histone H3 at Brachyury promoter is regulated by DETA/NO. ChIP assay followed by PCR to determine the occupancy of H3 acetylated at the Brachyury promoter. Primer set -231/-13 from AUG.

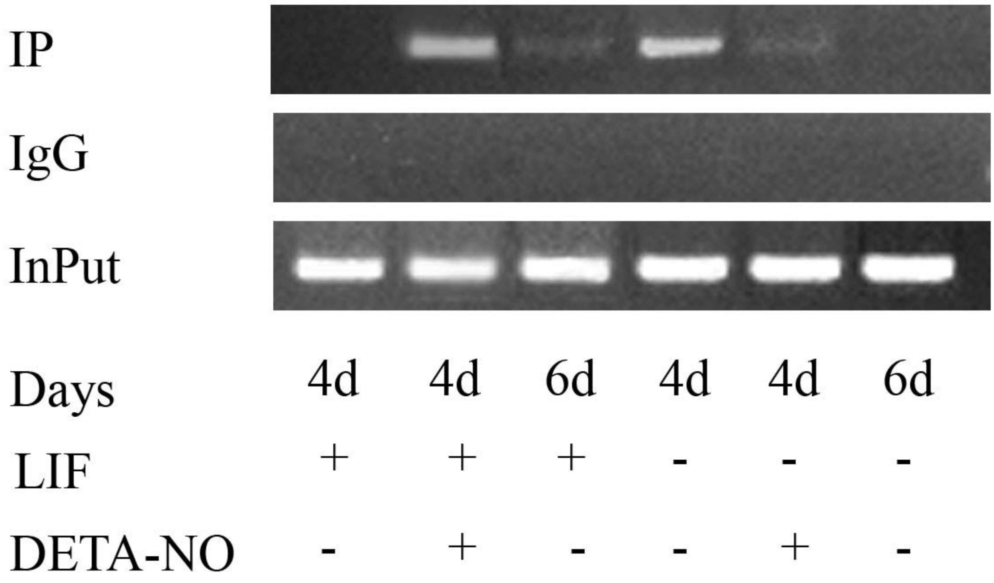

Supplement: Supplementary file 1 — The Supplementary Material shows the dynamics of reexpression of Nanog and Oct4 protein after DETA/NO challenge. In addition, a ChIP assay was performed to identify the occupancy of the H3 histone acetylated at Brachyury promoter in order to determine the regulation of this gene by nitric oxide. Antibodies and methodology used for both experiments is described in Section 2. [file 379678.f1.pdf]
